# Temporal variation in United States firearm injuries 1993-2008: results from a national data base

**DOI:** 10.5249/jivr.v6i1.351

**Published:** 2014-01

**Authors:** Randall T. Loder

**Affiliations:** ^a^Department of Orthopaedic Surgery, Indiana School of Medicine, Indiana University, and the James Whitcomb Riley Children’s Hospital, Indianapolis, Indiana, USA.

**Keywords:** Firearm, Injury, Temporal variation, Month, Weekday

## Abstract

**Background:**

There are few studies that address temporal variation in firearm associated injuries. It was the purpose of this study to analyze the temporal variation in the types and patterns of injuries associated with firearm use from a national data base.

**Methods:**

The database used was the Inter-University Consortium for Political and Social Research Firearm Injury Surveillance Study 1993-2008. Emergency department visits associated with firearm use were analyzed for month and day of the week for various demographic variables. Statistical analyses were performed using SUDAAN 10™ software to give national estimates. Temporal variation by month or day was assessed using histograms, circular distributions, and cosinor analyses. Variation by month and day combined were analyzed using three dimensional contours.

**Results:**

There were an estimated 1,841,269 injuries. Circular analyses demonstrated anon-uniform distribution for all parameters for both month and day of injury (p less than 0.001). The overall peak was September 15 with several exceptions. Injuries from BB guns had a peak on May 22, a diagnosis of a foreign body on July 11, and patients aged 10 to 14 years on April 9.The peak day was always Saturday/Sunday when significant variation existed. There were many different patterns for month and day combined. Some were “a rapidly rising high mountain starting at sea level” (hunting), or others a “series of mountain ranges starting from a high plain or steppe” (hospital admissions).

**Conclusions:**

This study provides altogether new information regarding temporal variation for injuries associated with firearms in the USA. These results can be used to assist medical resource allocation and prevention campaigns. Education campaigns can be emphasized before the peaks for which prevention is desired (eg. BB gun prevention campaigns should be concentrated in March, prior to the April/May peak).

## Introduction

Injuries due to firearms are a significant health burden in the United States of America. The number of studies addressing gunshot injuries is voluminous, but few^1-9^ address temporal variation of such injuries and none have mathematically modeled any temporal patterns when found. It was the purpose of this study to mathematically analyze the temporal variation in the types and patterns of injuries associated with firearm use from a national data base. This allows for a more general view, rather than a microscopic view. Such information can be used to assist health care providers and institutions caring for such patients to better allocate resources at both outpatient and inpatient levels. It can also guide appropriate timing for education and prevention campaigns in an effort to reduce these injuries. 

## Methods

The data for this study was obtained from the Inter-University Consortium for Political and Social Research Firearm Injury Surveillance Study 1993-2008 (ICPSR 30543)^10^ collected by the National Electronic Injury Surveillance System (NEISS). Further details regarding the acquisition of the ICPSR/NEISS data and guidelines for use of such data can be accessed from their respective web sites (ICPSR - www.icpsr.umich.edu, NEISS - www.cpsc.gov/library/neiss.html). 

The data for emergency department (ED) visits associated with firearm use from 1993 through 2008 was downloaded from the ICPSR website. It was analyzed for month and day of the week by hospital stratum, age, diagnosis, gender, race, marital status, type of firearm, perpetrator of injury, intent of injury (unintentional, assault, suicide, law enforcement), anatomic location of the injury, the geographic location of where the injury occurred, method of transportation to the ED, disposition from the ED, was the patient shot/not shot, and the involvement of drugs/crime/fight/ argument in the incident. (Not all firearm injuries occur when the person is shot, such as a firearm used as a blunt club in an assault, or a clavicle fracture from a rifle recoil). Race was classified according to Eveleth and Tanner^11^ as White, African, Amerindian (Hispanic and Native American) and Indo-Malay (Asian origins). The NEISS does not differentiate hospital locations by urban, suburban, rural; however, there are 5 strata designated by number of ED visits per year. These strata and their number of annual ED visits are: small (1-16,830), medium (16,831-28150), large (28,151-41,130), very large (> 41,130), and children’s hospitals (various numbers). Such strata can be viewed as a general proxy for rural (small), suburban (medium), and urban locations (large, very large).

Individual comments for each injury were searched to ascertain if either alcohol or hunting was involved. The detailed comments for each injury (recorded in the ICPSR data set) were searched using the FIND command in Microsoft Excel™ (Microsoft® Office 2003, Microsoft Corporation 1985-2003). The key words used to search for any injury involving alcohol were: alcohol, EtOH, intoxicated, drinking, drank, drunk, club, ethanol, saloon, tavern, liquor, booze, beer, whiskey, brandy, rum, vodka, scotch, tequila, wine, sake, champagne, and cognac. The key words used to search for any injury involving hunting were: hunt(ing), deer, elk, moose, bear, antelope, coyote, lion, wolf, boar, hog, groundhog, prairie dog, squirrel, rabbit, coon, beaver, waterfowl, goose/geese, turkey, duck, quail, coon, pheasant, bird, sparrow. 

As this is an exploratory study with no previous studies mathematically assessing the question of temporal variation in firearm associated injuries, there were no preconceived expectations. However, it could be postulated that there should be no difference in any monthly variation by gender, or race. Another hypothesis is that injuries sustained from shotguns and rifles, frequently used in hunting, would follow any patterns observed in the hunting group, while handguns, typically used in an assault situation would follow patterns similar to assaults. Regarding age, it is postulated that younger children would see an increase at the year end holiday when receiving firearms as presents, teenagers would demonstrate a peak in hunting season, young adults would show no significant variations due to them mostly being involved in violent events (assaults, crimes) which are likely to be uniform throughout the year, and that older adults would again demonstrate peaks during hunting season. Those injured by themselves or unintentional injuries would likely be hunting and show a similar pattern to hunting injuries. Suicides would likely be random with no temporal variation. Those injured on the street/ highway would likely be assaults and demonstrate similar patterns; those injured on farms would likely be due to hunting activities and should follow similar patterns; those injured at homes would be random. Rural injuries (small hospitals) are hypothesized to demonstrate similar patterns to these seen on farms or while hunting; assaults would show patterns seen in the urban (large and very large hospitals). It is postulated there will be an increase in injuries on the weekend, when more people are not working.

It was also desired to specifically explore patterns by the type of event (assault, unintentional) and by perpetrator. Such analyses might uncover differences in peaks by subgroups, and point towards specific potential preventive campaigns.

**Statistical Analyses**

Due to the stratified and weighted nature of the ICPSR data, statistical analyses were performed using SUDAAN 10™ software (RTI International, Research Triangle Park, North Carolina, 2008). This software accounts for the weighted and stratified nature of the data and calculates an estimated value across an entire population encompassed by the data set. In this case the ICPSR represents the entire United States of America. The estimated values of firearm injuries by month and year were so determined. 

**Figure 1:**
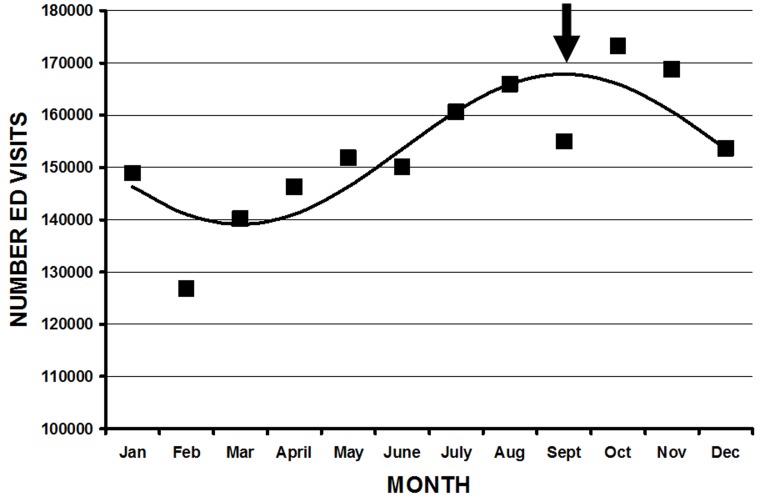
Cosinor analysis of all firearm injury ED visits (y axis) by month (x axis). The actual data is shown in the black squares, and the cosinor fit by the black line. This fit is represented by the equation. Number ED visits = 153490 + 14379cos((30t-15)-255), where t = 1 is January, 2 is February, etc, and was statistically significant (r^2^ = 0.69, p = 0.006). The peak is September 15 (arrow).

Several methods were used to analyze for temporal variation. The first was a simple review of the data as seen in histograms. This was then quantified using circular distribution which converts the data to a circular scale.^12^ The rectangular coordinates of an angle representing each month (360º/12 = 30º per month) or day (360º/72 = 51.4º per day) are calculated for each data point, using January as an angle of 0 and November as an angle of 330 and Sunday as an angle of 0 and Saturday as an angle of 308.4°. Each angle’s sine and cosine are represented on an x and y axis. From these data, the average month/day is the angle determined by the average of the sine and cosine components (angular mean), with a radius amplitude (r), and circular standard deviation or angular dispersion (s). The Rayleigh z-test was used to test for non uniformity in circular distribution. When the Rayleigh z-test demonstrated non uniformity in circular distribution, cosinor analysis^13-15^ was used to determine best fits for the data. Cosinor analysis is an extension of circular distribution and represents the mathematical best fit of the data to a cosine curve defined by the equation F(t) = M + Acos(ɷt + ɸ), where M = the mean level (termed mesor), A = the amplitude of the cosine curve, ɸ = acrophase (phase angle of the maximum value), ɷ = the frequency (which for monthly analysis is 360°/12 = 30° or for week day analysis is 360°/7 = 51.4°), and t = time (which in this case is each month or day). The overall p and r^2^ value for the goodness of fit of this equation were noted. When the p < 0.05, the data is not a uniform circular distribution but rather represents a rhythmic pattern described by the cosinor equation for M, A, and ɸ(Figure 1). The data were analyzed for the entire period of 12 months (monthly variation) and 7 days (weekday variation), as well as decreasing increments of 1 month or 1 day respectively. On occasion, the best monthly fit was not over a period of 12 months, but a different time span (eg. 5 or 6 months periodicity). Differences between statistically significant cosinor fits were assessed with the Bingham test.^16^ Cosinor analyses were performed with ChronoLab 3.0™ software (see Acknowledgement). For all analyses, a p <0.05 was considered statistically significant.

**Figure 2:**
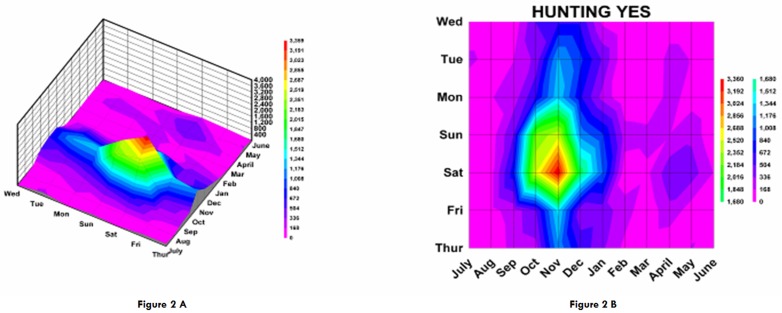
Examples of topographic contours representing month by day data. The number of ED visits are plotted onto a topographic “map” with the month on the x axis, the weekday on the y axis, and the number of ED visits on the z axis (or “elevation” of the contour). Twenty equal “contour elevations” were used to create the “topographic maps”. The lowest contour starting at 0 is purple/pink and the highest contour reaching the maximum number of patients is bright red. The data used in this figure is for those who were involved in hunting activities and sustained firearm injuries. Both three dimensional (A) and two dimensional (B) representations are shown.

Differences by both month and day were analyzed using a previously described method^17^ analogous to a topographical map with “contours of elevations”, since 3 dimensional cosinor analyses do not presently exist. The number of ED visits was plotted onto a topographic “map” with the month on the x axis, the weekday on the y axis, and the number of ED visits on the z axis (or “elevation” of the contour). Twenty equal “contour elevations” were used to create the “topographic maps”(Figure 2). These 3 dimensional topographic contours were created using DPlot Software 2.3.3.1 (Hyde Soft Computing LLC, Vicksburg, MS, 2001-2012) and subjectively reviewed to determine the “peaks” for each variable.

## Results

There were 61,419 firearm injuries in the ICPSR data base between 1993 and 2008, resulting in an estimated 1,841,269 injuries. Circular analyses demonstrated a non-uniform distribution for all parameters for both month and day of injury (all p <0.001). 

**General Exploratory Analyses**

Not all parameters demonstrated statistically significant fits using cosinor analysis for either month or weekday(Table 1). The overall peak month was September 15 (Figure 1). Although the majority of the peaks were in late summer and autumn, there were several exceptions. Injuries from BB guns had a peak on May 22, a diagnosis of a foreign body on July 11, and patients aged 10 to 14 years on April 9. There were several parameters with bimodal fits (two peaks throughout the year)(Figure 3A ,3B ). These were those injured at school/places of recreation ( May 5 and November 4), those injured during law enforcement activities (May 1 and October 31), those whose ages were 45 to 54 years (May 21, November 19), > 55 years (May 14, November 12), and > 65 years (May 16, November 14). There were other parameters that had significant cosinor fits, but such fits were not visually the best model due to a particular higher peak (eg. hunting, rifles, shotguns) (Figure 4). The overall peak day was on the weekend, usually at 2400 Saturday/0000 Sunday for those parameters that demonstrated a significant fit. There was no weekday pattern for those who were divorced/separated, injured during law enforcement activities, or suicides. 

**Table 1:** Cosinor analyses by month and weekday for an estimated 1,841,269 firearm injuries 1993-2008 using the ICPSR national data base

Parameter	n	Month	Weekday		Parameter	n	Month	Weekday
All	1841359	15-Sep	Sat/Sun		Incident Type			
Gender					Unintentional	530023	24-Nov	Sat/Sun
Male	1593448	17-Sep	Sat/Sun		Assault	955359	17-Aug	Sat/Sun
Female	247256	24-Aug	Sat/Sun		Suicide	92623	-	Sat/Sun
Race					Law Enforcement	21368	-	-
White	662276	4-Nov	Sat/Sun		Marital Status			
African	634078	11-Aug	Sat/Sun		Never	669628	1-Sep	Sat/Sun
Amerindian	254552	-	Sat/Sun		Married	228859	3-Nov	Sat/Sun
Asian	20406	-	Sat/Sun		Divorce/Sep	44919	29-Aug	Sat/Sun
Firearm					Other	24238	12-Aug	-
Handgun	498848	1-Oct	Sat/Sun		Disposition from ED			
Rifle	109679	7-Nov	-		Released	1181291	20-Sep	Sat/Sun
Shotgun	103475	30-Oct	Sat/Sun		Admitted	555659	1-Sep	Sat/Sun
BB	354776	22-May	Sat/Sun		Fatality	95222	24-Aug	Sat/Sun
Diagnosis					Argument			
Contusion/Abrasion	114995	-	Sat/Sun		Yes	121885	27-Sep	Sat/Sun
Foreign Body	244095	11-Jul	Sat/Sun		No	626144	8-Nov	Sat/Sun
Laceration	103558	25-Oct	Sat/Sun		Crime			
Puncture	245317	18-Aug	Sat/Sun		Yes	238628	6-Sep	Sat/Sun
Internal Injury	66120	5-Sep	Sat/Sun		No	619793	9-Nov	Sat/Sun
Fracture	695964	29-Sep	Sat/Sun		Drugs			
Anatomic Area					Yes	58453	-	-
Head/Neck	543507	26-Oct	-		No	625734	15-Nov	Sat/Sun
Upper Trunk	268025	10-Aug	-		Fight			
Lower Trunk	199054	12-Aug	Sat/Sun		Yes	149301	8-Sep	Sat/Sun
Arm/Hand	341952	17-Aug	Sat/Sun		No	651145	8-Nov	Sat/Sun
Leg/Foot	444352	30-Aug	Sat/Sun		Rape			
Location					Y	9089	-	-
Home	498935	7-Nov	Sat/Sun		N	1822270	15-Sep	Sat/Sun
School/Rec	51301	-	-		Shot			
Strt/Hghwy	305153	17-Aug	Sat/Sun		Y	1418741	11-Aug	Sat/Sun
Other Prop	202665	22-Oct	Sat/Sun		N	432168	19-Oct	Sat/Sun
Farm	6071	22-Nov	-		EtOH			
ED Transport					Y	9089	20-Sep	Sat/Sun
EMS	398662	29-Aug	Sat/Sun		N	1822270	20-Sep	Sat/Sun
Air	286959	17-Sep	Sat/Sun		Hunting			
Priv Vehic	144053	18-Aug	Sat/Sun		Y	35965	17-Nov	Sat/Sun
Walk In	44059	-	Sat/Sun		N	1805839	1-Sep	Sat/Sun
Police	520716	28-Oct	Sat/Sun		Age Group (years)			
Other	45311	5-Aug	Sat/Sun		0 to 4	13969	-	Sat/Sun
Hospital Stratum					5 to 9	56462	-	Sat/Sun
Small	377098	-	Sat/Sun		10 to 14	173035	9-Apr	Sat/Sun
Medium	317928	-	Sat/Sun		15 to 19	382684	28-Aug	Sat/Sun
Large	493311	31-Aug	Sat/Sun		15 to 24		1-Sep	Sat/Sun
Very Large	634992	5-Sep	Sat/Sun		20 to 24	365909	6-Sep	Sat/Sun
Children’s	17940	9-July	Sun		25 to 34	406923	28-Sep	-
Perpetrator					35 to 44	231299	2-Oct	Sat/Sun
Stranger	469554	8-Sep	Sat/Sun		45 to 54	110784	-	Sat/Sun
Self	469554	18-Nov	Sat/Sun		55 to 64	50101	-	Sun
Friend/Acq	138549	-	Sun		65+	39765	-	-
Spouse/Ex	13589	-	Sat					
Other Relative	55904	-	-		0 to 14	243458	-	Sat/Sun
Not Seen/Other	241669	29-July	Sat/Sun		15 to 34	1155674	8-Sep	Sat/Sun
					35 to 54	342083	8-Oct	Sat/Sun
					55+	89866	17-Oct	-

**Figure 3A:**
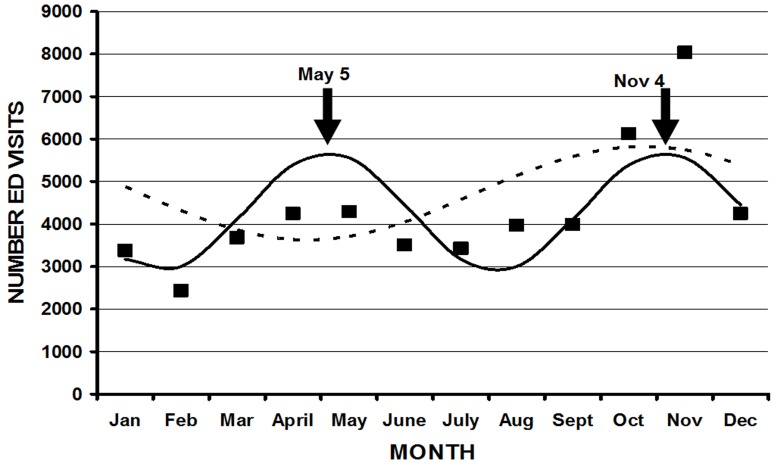
Injuries occurring at school/places of recreation demonstrated a statistically significant bimodal variation (solid line) with a periodicity of 6 months, represented by the equation: Number ED visits = 4278 + 1380cos(60t - 247), r^2^ = 0.49, p = 0.049, where t = the month of injury (1 = January, 2 = February, 3 = March, 4 = April, 5 = May, 1 = June, 2 = July, etc). The closed squares represent the actual number of ED visits per month. A unimodal fit (hatched line), represented by the equation: Number ED visits = 4275 + 1099cos(30t - 293) (r2 0.31, p = 0.19), was not statistically significant. The two peaks in the bimodal model correspond to May 5 and November 4 (solid arrows).

**Figure 3B:**
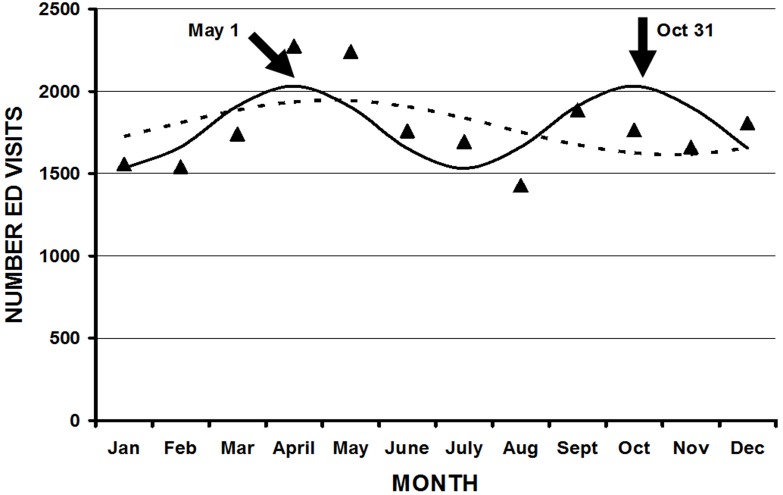
Injuries due to law enforcement activity demonstrated a statistically significant bimodal variation (solid line) with a periodicity of 6 months, represented by the equation: Number ED visits = 1782 + 249cos(60t - 239), r^2^ = 0.51, p = 0.039. The closed triangles represent the actual number of ED visits per month. A unimodal fit (hatched line), represented by the equation: Number ED visits = 1781 + 165cos(30t - 125) (r2 0.23, p = 0.31), was not statistically significant. The two peaks in the bimodal model correspond to May 1 and October 31 (solid arrows).

**Figure 4:**
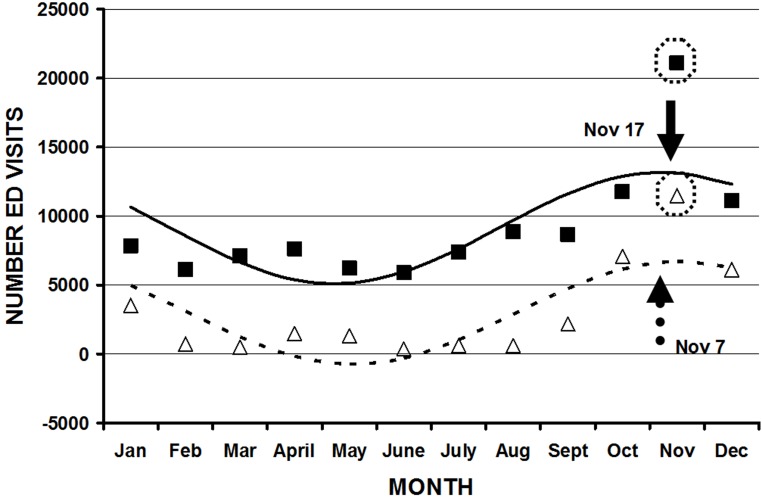
Cosinor fits for those injured with rifles (black squares and solid black line) and while hunting (open triangles and hatched line). There were statistically significant fits for both rifles (Number ED visits = 9136 + 4035cos((30t-15)-307), r^2^ = 0.50, p = 0.042, peak November 17) and hunting (Number ED visits = 2991+ 3723cos((30t-15)-317), r2 = 0.62, p = 0.013, peak November 7) although the month of November was a significant outlier for both (outlined by the dotted octagon).

**Figure 5:**
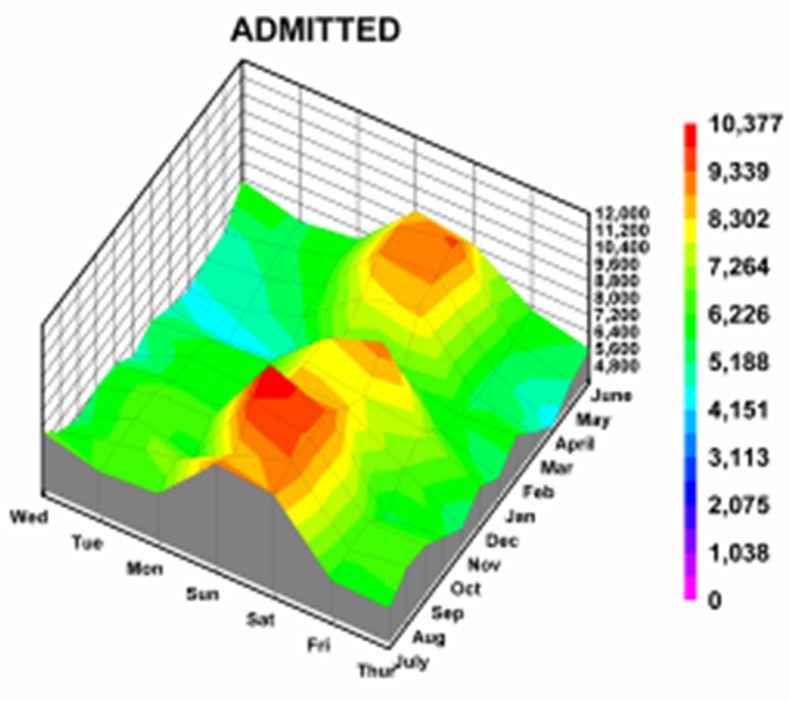
Three dimensional topographic contour for those who were admitted to the hospital for injuries associated with a firearm. Note the starting level of 4800 patients “medium altitude plain” with various “rising mountain ranges”, the tallest one at 10,377 patients (Saturday/Sunday in October/November), but also with 2 other peaks (Saturday in January, and Saturday/Sunday in May/June).

Month by weekday topographic maps demonstrated many different patterns. Some were “a rapidly rising high mountain starting at sea level” such as with hunting activities (Figure 2), or others demonstrating a “series of mountain ranges starting from a high plain or steppe” (Figure 5). The peak tabulations for month and day (upper 2 levels of red) are given in Table 2, and various examples are graphically shown in Figure 6.

**Table 2:** Compilation of weekday by month topographic analyses of firearm injuries

Parameter	Peak 1	Peak 2		Parameter	Peak 1	Peak 2
All	Sat-Sun, Oct			Incident Type		
Gender				Unintentional	Sat-Sun, Sep-Nov	
Male	Sat-Sun, Oct			Assault	Sat-Sun, July-Nov	
Female	Sun, Oct			Suicide	Mon, Nov	Tue, Jan^
Race				Law Enforcement	Thur, May	Sat, Jun
White	Sat, Oct			Marital Status		
African	Sat-Sun, July-Oct	Sat-Sun, May		Never	Sat-Sun, Oct	
Amerindian	Sat-Sun, Oct	Sat, July		Married	Sat-Sun, Oct-Nov	
Indo-Malay	Sun, Jan	Sat, Sept		Divorce/Sep	Sun-Tues, Sep-Nov	
Firearm				Other	July, Wed	
Handgun	Sat-Sun, Aug-Nov			Disposition from ED		
Rifle	Sat, Nov			Released	Sat-Sun, Sep-Nov	
Shotgun	Sat-Sun, Oct			Admitted	Sat-Sun, Aug-Sep	Sat, May
BB	Sat-Sun, April			Fatality	Sat-Sun, Oct	
Diagnosis				Argument		
Contusion/Abrasion	Sat-Sun, Sep-Nov			Yes	Sat-Sun, Aug-Nov	
Foreign Body	Sun, Oct			No	Sat, Nov	
Laceration	Sat-Sun, Nov			Crime		
Puncture	Sat-Sun, Oct			Yes	Fri-Mon, July-Oct	
Internal Injury	Sun, Feb			No	Sat, Nov	
Fracture	Sat-Sun, Sep			Drugs		
Anatomic Area				Yes	Sat-Mon, Oct	Sat, April-May
Head/Neck	Sat-Sun, Oct-Nov			No	Sat, Nov	
Upper Trunk	Sat, Oct			Fight		
Lower Trunk	Sat, Oct	Sun, May		Yes	Sun, Oct	
Arm/Hand	Sun, Sep	Sun, Mar		No	Sat, Nov	
Leg/Foot	Sat-Sun, Oct	Sat-Sun, Jan		Rape		
Location				Y	Sat, April	
Home	Sat-Sun, Nov			N	Sat-Sun, Sep-Nov	
School/Rec	Sat-Sun, Nov			Shot		
Strt/Hghwy	Sat-Sun, Oct			Y	Sat, June	Sun, Oct
Other Prop	Sat-Sun, Oct			N	Sat-Sun, Oct-Nov	
Farm	Fri-Sat, Nov			EtOH		
ED Transport				Y	Sat-Sun, Sep-Oct	
EMS	Sat-Sun, Aug-Oct			N	Sat-Sun, Sep-Nov	
Air	Sat-Sun, Aug	Sat-Sun, Oct		Hunting		
Priv Vehic	Sat, May	Sun, Oct		Y	Sat, Nov	
Walk In	Sun-Mon, July	Sat, Nov^		N	Sat-Sun, Sep-Nov	
Police	Sun, Oct			Age Group (years)		
Other	Sun, Nov			0 to 4	Sun, Oct	
Hospital Stratum				5 to 9	Sat-Sun, Jan	
Small	Sat-Sun, Oct-Nov			10 to 14	Sun, Mar	Sat, Nov
Medium	Sat-Sun, July-Dec	Sat-Sun, Mar-June		15 to 19	Sun, Nov	Sun, Aug
Large	Sat-Sun, Sep-Nov			15 to 24	Sun, Oct	
Very Large	Sat-Sun, June-Sep			20 to 24	Sat-Sun, Sep-Nov	
Children’s	Fri-Wed, July-Sep	Sat-Sun, May-June		25 to 34	Sat-Sun, Aug-Nov	
Perpetrator				35 to 44	Sat-Sun, Oct-Nov	
Stranger	Sat-Sun, Aug-Sep	Sat-Sun, Dec and May		45 to 54	Sun, Nov	
Self	Sat-Sun, Nov			55 to 64	Sun, Oct-Dec	
Friend/Acq	Sat-Sun, Oct	Sat, Jan		65+	Sat, Nov	
Spouse/Ex	Sat-Sun, Sep					
Other Relative	Sat-Sun, Oct-Dec			0 to 14	Sat-Sun, Oct-April	
Not Seen/Other	Sat-Sun, July-Nov	Sat-Sun, Jan and June		15 to 34	Sat-Sun, Sep-Nov	
				35 to 54	Sat-Sun, Sep-Nov	
				55+	Sat-Mon, Sep-Dec	

^ there were additional peaks for Walk In, peak 3 was Sunday, June; peak 4 was Monday, April for Suicide, peak 3 was Sunday, February

Figure 6: Variations in firearm both month and weekday as shown on two dimensional topographic contour representations.

**A:**
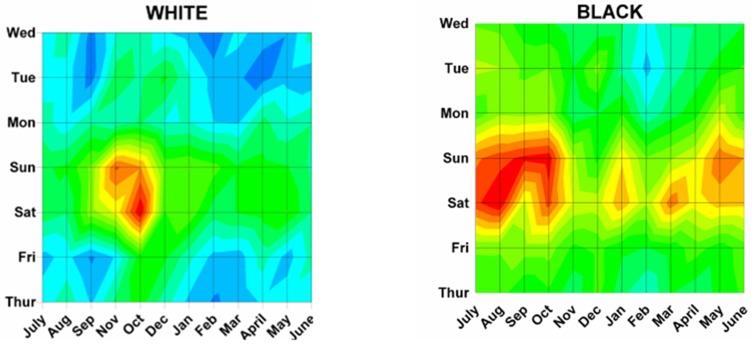
Whites and Africans.

**B:**
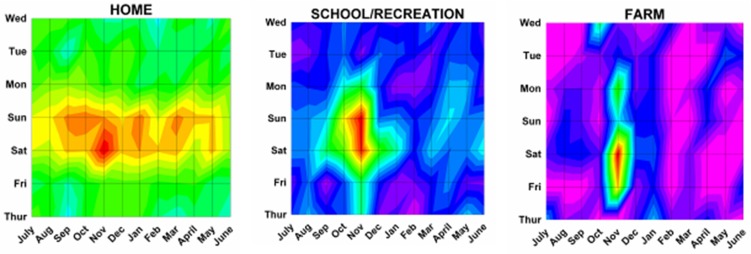
Geographic location of injury.

**C:**
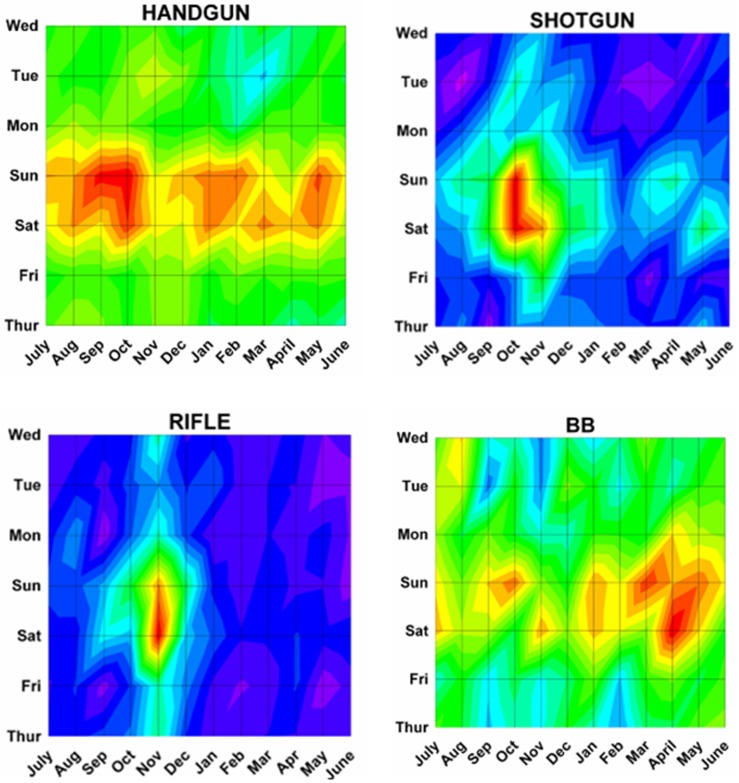
By firearm type.

**D:**
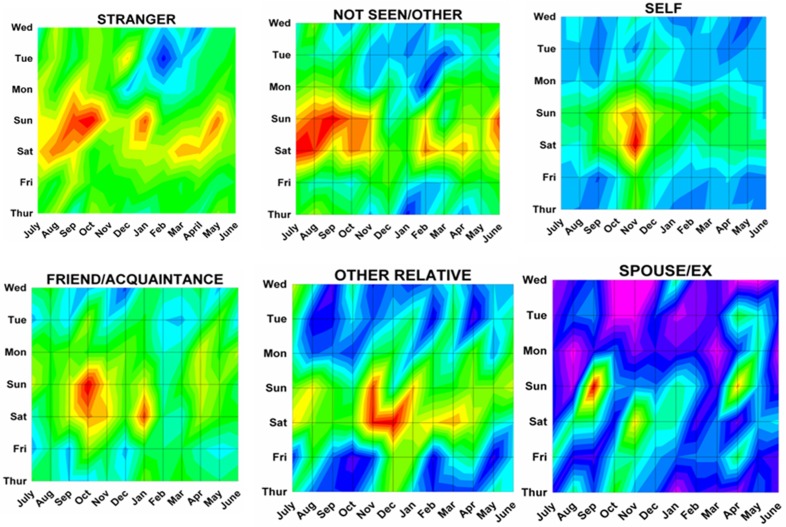
By perpetrator.

**E:**
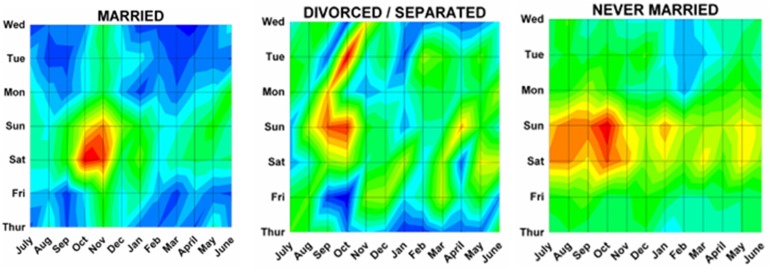
By marital status

**F:**
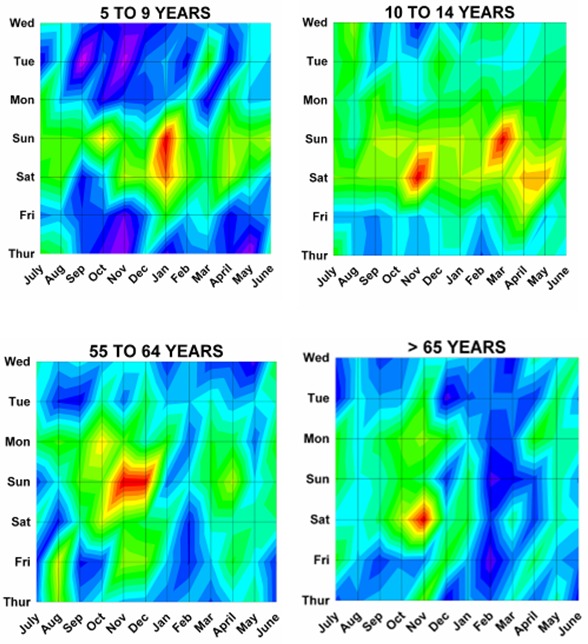
By age group.

**G:**
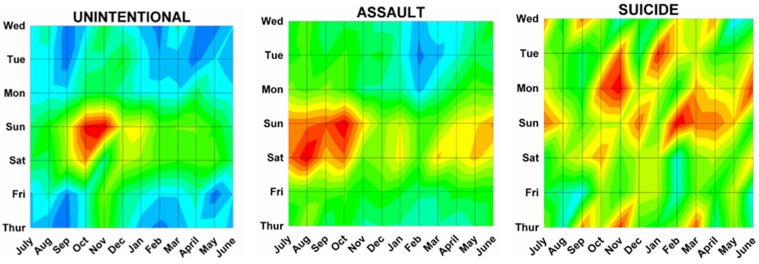
By incident type.

**H:**
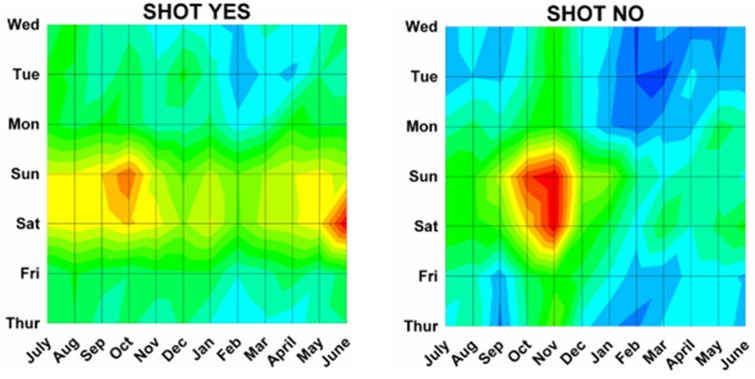
Shot and not shot.

**I:**
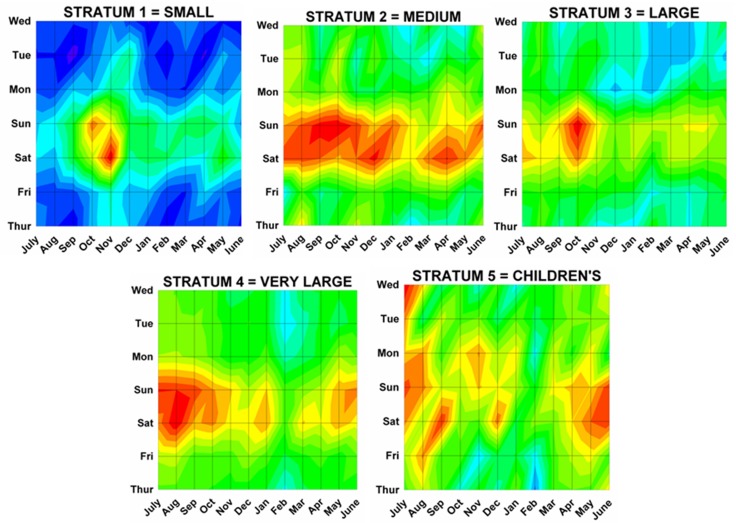
By hospital stratum.

**Subgroup Patterns by Incident Type and Perpetrator**

The detailed results for month are shown in Table 3. Injuries from suicides and law enforcement, and when the perpetrator was a spouse/ex or other relative were excluded due to small numbers. Unintentional injuries were predominantly in the autumn months, and assaults in the summer; notable exceptions for unintentional injuries were BB guns with a peak in April, those occurring at home in January, and those in the 0 to 14 year age group in February. Nearly all the self inflicted injuries occurred in the autumn months regardless of subgroup, those by strangers in the late summer/early autumn, and in the summer when the perpetrator was not seen. All significant weekday results always occurred on the weekends; thus no detailed results are shown.

**Table 3:** Subgroup cosinor analyses by month

	Unintentional	Assault	Stranger	Self	Friend/Acq	Not Seen/Other
Gender						
Male	24-Nov	17-Aug	14-Sep	26-Nov	22-May, 21-Oct	28-July
Female	23-Oct	17-Aug	7-Sep	-	-	-
Race						
White	23-Nov	3-Sep	18-Sep	22-Nov	25-May, 21-Oct	-
African	-	4-Sep	7-Sep	-	-	20-July
Amerindian	29-Jan	9-Sep	-	3-Feb, 3-Nov	-	7-Aug
Asian	-	14-Jan, 14-Sep	-	-	-	-
Firearm						
Handgun	16-Jan, 18-Nov	30-Aug	11-Oct	22-Jan, 22-Nov	30-Aug	-
Rifle	8-Nov	-	-	9-Nov	5-Oct	-
Shotgun	14-Nov	4-Aug	1-Sep	20-Nov	27-May, 26-Oct	26-Apr, 25-Sep
BB	17-Apr	2-July	11-Jun	-	-	1-July
Anatomic Area						
Head/Neck	21-Nov	8-Sep	3-Oct	14-Nov	-	22-Aug
Upper Trunk	16-May, 15-Nov	7-Aug	-	-	-	-
Lower Trunk	7-May, 6-Nov	12-Aug	-	-	-	12-Aug
Arm/Hand	7-May, 6-Nov	5-Aug	22-July	7-May, 6-Nov	-	15-July
Leg/Foot	12-Dec	14-Aug	11-Sep	13-Dec	-	26-July
Location						
Home	2-Jan	4-Sep	23-Nov	5-Dec	-	30-July
School/Rec	13-Nov	30-June	13-July	12-Nov	-	5-Sep
Strt/Hghwy	-	16-Aug	22-Aug	-	-	29-July
Other Prop	5-Nov	-	-	6-Nov	9-Nov	5-Mar, 5-July, 4-Nov
Farm	23-Nov	-	-	-	-	-
Hospital Stratum						
Small	22-Nov	23-July	-	24-Nov	25-May, 24-Oct	15-May, 16-Nov
Medium	-	11-Sep	15-Sep	-	-	10-Aug
Large	14-Nov	11-Aug	30-Aug	-	-	13-July
Very Large	1-April, 1-Aug, 1-Dec	16-Aug	13-Sep	-	5-Aug	29-July
Children’s	-	29-July	-	12-Sep	-	6-July
Shot						
Yes	23-Dec	12-Aug	28-Aug	-	-	26-July
No	8-Nov	7-Sep	2-Oct	7-Nov	-	-
EtOH						
Yes	-	2-Sep	20-Sep	-	5-Sep	-
No	22-Nov	16-Aug	6-Sep	18-Nov	-	28-July
Hunting						
Yes	24-Nov	8-Nov	13-Sep	-	26-Nov	16-Oct
No	18-Nov	18-Aug	7-Sep	19-Nov	-	-
Age Group (years)						
0 to 14	15-Feb	19-Jun	30-Jun	17-Mar	-	24-Jun
15 to 34	17-Nov	16-Aug	14-Sep	12-Nov	23-May, 22-Oct	5-Aug
35 to 54	8-Nov	4-Sep	-	12-Nov	7-Oct	21-Aug
55+	12-May, 10-Nov	23-Aug	3-Oct	14-May, 13-Nov	-	28-May

When there are 2 or more dates, then the best cosinor fit was bi/multimodal. Dates in italics are for fits with 0.05 p < 0.10.

## Discussion

Limitations of the study must first be acknowledged. One limitation of the NEISS data is that it only identifies those individuals who sought care in the ED. It does not include those who might have been treated in urgent care centers, physician offices, or those patients who did not seek medical care. The overall number of injuries in this study is therefore lower than the real number of injuries. The NEISS is thus skewed to more serious injuries, since patients sustaining significant injuries will likely seek immediate care in the ED. Another potential limitation is the accuracy of the NEISS data. However it appears to have an accuracy of at least mid 90%. ^18,19^ With small numbers of individuals per category, the estimated national number estimates may be subject to some inaccuracy. With these caveats in mind, this new detailed background data can serve as a reference for future studies regarding the temporal variation of injuries associated with firearms.

This is the first study using a large national data base to determine temporal variations in injuries from firearm use. There are several studies that have looked at this question in their local area, but none with any mathematical modeling. In Pretoria, South Africa^3^ there were peaks in March and September. In Finland,^4^ there was a peak in September and October with no mention of the weekday. In Manchester, England^5^ there was no pattern regarding day of injury. In Illinois,^2^ assaults from firearm injuries demonstrated a peak on Fridays.

In a study of stray bullet shootings in the United States,^6^ there was a peak in the summer and on Thursdays. Gunshot injuries in south central Los Angeles^7^ were more frequent in the summer months; in Africans, there was uniform distribution throughout the week, while for Hispanics they were concentrated on Saturday through Monday. Craniofacial gunshot wound injuries in Tehran, Iran^8^ had a peak in January, with no mention of the weekday. In children 0 to 19 years old from East Baton Rouge, Louisiana, there were peaks in the summer and December; 24% occurred on Friday and 20% on Sunday.^1^ In Alabama children^20^ a peak was noted in the 2nd half of the year (July through December). In 749 children from the National Pediatric Trauma Registry^9^ 156 (53.4%) of unintentional injuries occurred Friday-Sunday; no monthly data was given.

Other studies assess only fatalities. Accidental firearm fatalities in Tennessee children and teenagers^21^ demonstrated two peaks, one in November and one June; rural deaths were more common in November while urban deaths were more common in June. Most accidental firearm hunting deaths in Sweden ^22^ occurred September through December and on Saturday/Sunday. A study of 444 firearm fatalities in Diyarbakir, Turkey^23^ demonstrated that there were more homicides in spring and summer, while there were more suicides and accidental deaths only in the spring. In Cagliari, Italy ^24^ firearm suicides were the highest in February with no variation in day of the week. In Bari, Italy,^25^ 28 of 82 suicides from firearms occurred in November through January, although there was no real pattern. In three United States cities (Allentown, Pennsylvania; Youngstown, Ohio; Cedar Rapids, Iowa)^26^ the number of suicides were relatively equal October to March and April to September; 63-72% of the suicides occurred on the weekdays and the remainder on weekends.

The data from several of these studies in the literature, where available, was extracted and subjected to cosinor analysis (Table 4). For monthly variation, the peak was September 26 in Finland ^4^ for all injuries and Sept 27 for unintentional injuries. In Los Angeles^7^ the peak for all patients was July 4 and for Africans June 27. Hunting deaths in Sweden^22^ had a peak on October 6. Head injuries from firearms in Iran^8^ demonstrated a peak on December 30. There was no difference by the Bingham test between Finland and our USA data set for all injures, but there was a difference in unintentional injuries (Sep 27 vs Nov 24, p = 0.018). Similarly the data from Los Angeles was different than the USA data set for all injuries (July 4 vs Sept 16, p = 0.007) and Africans (June 27 vs Aug 11, p = 0.029). There was a difference in head injuries between Iran and the USA data (Dec 30 vs Oct 26, p = 0.001). Those injured by stray bullets in the USA^6^ or children 0 to 19 years of age in East Baton Rouge, Louisiana^1^ demonstrated no significant cosinor fit. For weekday variation, the only significant cosinor fit was for the Hispanic patients in southern Los Angeles (Sunday, p = 0.01), which was not statistically different from the Saturday/Sunday in our Amerindian group (Bingham test, p = 0.14).

**Table 4:** Cosinor analyses on literature data

Geographic Location	Study	p value	r^2^	Mesor	Amp	ɸ	Date/Weekday
Monthly Data							
Finland	Matilla (4)						
All		0.006	0.68	209	32	265	26-Sep
Assault		0.063	0.46	52.2	6.7	33	-
Self Inflicted		0.32	0.23	45.1	3.4	293	-
Unintentional		0.001	0.78	91.7	27.6	266	27-Sep
Unknown		0.79	0.05	19.7	2.1	302	-
Los Angeles, CA	Weaver (7)						
All		0.005	0.69	63.7	18.4	182	4-Jul
African		0.007	0.67	41.1	12	176	27-Jun
Hispanic		0.13	0.23	22.6	6.6	193	-
Sweden Hunting Deaths	Junuzovic (22)	0.095	0.61	4.75	5.22	275	6-Oct
USA - Stray Bullets	Wintemute (6)	0.076	0.44	23.7	5.9	189	-
Iran - Head	Taher (8)	<0.0001	0.80	94.1	122.1	359	30-Dec
East Baton Rouge, LA 0 to 19 yrs	Ary (1)	0.079	0.43	38.5	9.3	153	-
Weekday Data							
Los Angeles, CA	Weaver (7)						
All		0.37	0.40	108	11.7	4	-
Black		0.22	0.53	72.6	11.9	234	-
Hispanic		0.01	0.89	35.3	21.4	29	Sunday
East Baton Rouge, LA 0 to 19 yrs	Ary (1)	0.96	0.021	66.4	4.8	271	-
Sweden Hunting Deaths	Junuzovic (22)	0.13	0.65	6.7	3.9	28	-
USA - Stray Bullets	Wintemute (6)	0.46	0.33	40.4	5.3	300	-

Several of our hypotheses proved true, while others did not. It was hypothesized that there would be more injuries on the weekend when people were not working, which was statistically confirmed by a peak at 2400 Saturday/0000 Sunday. As postulated there were no differences in monthly peaks between males and females (Sep 17 and Aug 24, p = 0.17). We postulated no differences by race; however there were differences between Whites and Africans (Nov 4 vs Aug 11, p = 0.013). As postulated injuries from rifles and shotgun had peaks on Nov 7 and Oct 30, similar to hunting peak on Nov 17 (rifle vs hunting p = 0.19, shotgun vs hunting p = 0.43). It was hypothesized that handguns would have a peak similar to that of assaults; however, that was not so, with the peaks for assaults being Aug. 17 and handguns Oct 1 (p = 0.012). When looking at different age groups, the hypothesis that younger children would have a year end peak due to the holiday season was not seen as there was no significant peak for those 0 to 14 years old. Teenagers 15 to 19 years of age did demonstrate a peak of Aug 28, but still different from the postulated hunting peak of Nov 17 (p = 0.015). The hypothesis that young adults would demonstrate no significant monthly variation as most would be violent events, which would likely be uniform throughout the year, was not proven; those 15 to 34 years of age had a peak of Sep 8, and those involved in a crime had a peak of Sep 6. Older adults demonstrated a peak similar to those with hunting injuries (> 55 years – Oct 17, hunting – Nov 17, p = 0.33). Regarding incident types, there was no peak for suicides, as postulated. Unintentional injuries were confirmed to show patterns similar to hunting (Nov 24 vs Nov 17, p = 0.80). Those injured on street/highways were postulated to be similar to assaults which was confirmed with street/highways and assaults both on Aug 17. The hypotheses that those injured on farms would be similar to those involved in hunting activities was confirmed (Nov 22 vs Nov 17, p = 0.91). Those injured at home were not random as postulated, but had a peak on Nov 7. It was confirmed that those injured by themselves were similar to hunting (Nov 18 vs Nov 17, p = 0.85) and unintentional injuries (Nov 18 vs Nov 24, p = 0.80). It was postulated that small hospitals would have peaks similar to hunting and farm injuries; however small hospitals demonstrated no monthly pattern. It was confirmed that the peak for assaults were similar to large (Aug 17 vs Aug 3, p = 0.40) and very large (Aug 17 vs Sep 5, p = 0.97) hospitals.

Analyses within the subgroups demonstrated interesting findings. Those injured at home in aggregate demonstrated a peak on Nov 7; however unintentional home injuries peaked Jan 2 while assaults were Sep 4 (p = 0.001). Those who were shot had an overall peak on Aug 11; but those who were shot unintentionally had a peak on Nov 14 and those who were assaulted Aug 12 (p <0.0001). Those who were 0 to 14 years of age had no peak in aggregate; however those 0 to 14 years of age who were assaulted had a peak on June 19 and those with unintentional injuries a peak on Feb 15 (p = 0.004). Unintentional injuries from rifles were Nov 8, shotguns Nov 14, and BB guns April 17 (rifle vs BB p = 0.004, shotgun vs BB p = 0.002). Self inflicted injuries for those 0 to 14 years of age were March 17 and 15 to 34 years Nov 12 (p = 0.004); unintentional injures for those 0 to 14 years of age were Feb 15, 15 to 34 years Nov 17, and 35 to 54 years Nov 8 (0 to 14 vs 15 to 34 years, p = 0.035; 0 to 14 vs 35 to 54 years, p = 0.017). Large hospitals had an overall peak on Aug 31; unintentional injuries seen at large hospitals peaked Nov 14 while assaults peaked Aug 11 (p = 0.002). 

Our results can be used to assist in both allocations of resources for medical institutions caring for these injuries as well as guide prevention campaigns. Education campaigns for unintentional and self inflicted injuries can be postulated; those for assaults and injuries caused by strangers or those not seen are more difficult to postulate. For instance, public education service announcements targeting accidental injuries from BB guns could be concentrated in March, prior to the April peak. Education for hunters and users of rifles and shotguns to prevent unintentional injuries could be concentrated in the late summer. Those who live in their own homes will need reminders in the last part of the year to influence unintentional injuries. Children 5 to 9 years of age had a peak in January; parental education campaigns regarding firearm safety could be concentrated in December. The peaks for hospital admission were on weekends in the autumn and spring; this can help guide institutions in allocation of staffing and other resources(Figure 5). It needs to be remembered there are variations by hospital size; this study that demonstrated no monthly variation for small or medium sized hospitals, but with Aug/Sep peaks for large and very large hospitals and July peak for children’s hospitals. There may also be other local variations in these temporal patterns, as noted by the differences when comparing the results of cosinor analysis from the literature compared to the overall USA data set. Nevertheless this study gives significantly new information regarding temporal variation in injuries from firearms in the USA at the national level. 
